# Metabolic Disorders Are Associated With Drug-Induced Liver Injury During Antituberculosis Treatment: A Multicenter Prospective Observational Cohort Study in Korea

**DOI:** 10.1093/ofid/ofad422

**Published:** 2023-08-07

**Authors:** Jihye Lim, Ju Sang Kim, Hyung Woo Kim, Yong Hyun Kim, Sung Soo Jung, Jin Woo Kim, Jee Youn Oh, Heayon Lee, Sung Kyoung Kim, Sun-Hyung Kim, Jiwon Lyu, Yousang Ko, Sun Jung Kwon, Yun-Jeong Jeong, Do Jin Kim, Hyeon-Kyoung Koo, Yangjin Jegal, Sun Young Kyung, Tai Joon An, Jinsoo Min

**Affiliations:** Division of Gastroenterology and Hepatology, Department of Internal Medicine, Yeouido St. Mary's Hospital, College of Medicine, The Catholic University of Korea, Seoul, Republic of Korea; Division of Pulmonary and Critical Care Medicine, Department of Internal Medicine, Incheon St. Mary's Hospital, College of Medicine, The Catholic University of Korea, Seoul, Republic of Korea; Division of Pulmonary and Critical Care Medicine, Department of Internal Medicine, Incheon St. Mary's Hospital, College of Medicine, The Catholic University of Korea, Seoul, Republic of Korea; Division of Pulmonary, Allergy, and Critical Care Medicine, Department of Internal Medicine, Bucheon St. Mary's Hospital, College of Medicine, The Catholic University of Korea, Seoul, Republic of Korea; Division of Pulmonary and Critical Care Medicine, Department of Internal Medicine, Chungnam National University Hospital, Daejeon, Republic of Korea; Division of Pulmonary and Critical Care Medicine, Department of Internal Medicine, Uijeongbu St. Mary's Hospital, College of Medicine, The Catholic University of Korea, Seoul, Republic of Korea; Division of Pulmonary, Allergy, and Critical Care Medicine, Department of Internal Medicine, Korea University Guro Hospital, Korea University College of Medicine, Seoul, Republic of Korea; Division of Pulmonary, Critical Care, and Sleep Medicine, Department of Internal Medicine, Eunpyeong St. Mary's Hospital, College of Medicine, The Catholic University of Korea, Seoul, Republic of Korea; Division of Pulmonary and Critical Care Medicine, Department of Internal Medicine, St. Vincent's Hospital, College of Medicine, The Catholic University of Korea, Seoul, Republic of Korea; Division of Pulmonary and Critical Care Medicine, Department of Internal Medicine, Chungbuk National University Hospital, Cheongju, Republic of Korea; Department of Pulmonary and Critical Care Medicine, Soonchunhyang University Cheonan Hospital, Soonchunhyang University College of Medicine, Cheonan, Republic of Korea; Division of Pulmonary, Allergy, and Critical Care Medicine, Department of Internal Medicine, Kangdong Sacred Heart Hospital, Hallym University College of Medicine, Seoul, Republic of Korea; Division of Pulmonary and Critical Care Medicine, Department of Internal Medicine, Konyang University Hospital, Konyang University College of Medicine, Daejeon, Republic of Korea; Division of Pulmonary and Critical Care Medicine, Department of Internal Medicine, Dongguk University Ilsan Hospital, Goyang, Republic of Korea; Division of Allergy and Respiratory Medicine, Soonchunhyang University Bucheon Hospital, Soonchunhyang University College of Medicine, Bucheon, Republic of Korea; Division of Pulmonary and Critical Care Medicine, Department of Internal Medicine, Ilsan Paik Hospital, Inje University College of Medicine, Goyang, Republic of Korea; Division of Pulmonary and Critical Care Medicine, Department of Internal Medicine, Ulsan University Hospital, Ulsan University College of Medicine, Ulsan, Republic of Korea; Division of Pulmonology, Departments of Internal Medicine, Gachon University Gil Hospital, Incheon, Republic of Korea; Division of Pulmonary and Critical Care Medicine, Department of Internal Medicine, Yeouido St. Mary's Hospital, College of Medicine, The Catholic University of Korea, Seoul, Republic of Korea; Division of Pulmonary and Critical Care Medicine, Department of Internal Medicine, Seoul St. Mary's Hospital, College of Medicine, The Catholic University of Korea, Seoul, Republic of Korea

**Keywords:** antitubercular agents, chemical and drug-induced liver injury, metabolic syndrome, risk factors, tuberculosis‌

## Abstract

**Background:**

Drug-induced liver injury (DILI) may lead to the discontinuation of antituberculosis (anti-TB) treatment (ATT). Some studies have suggested that metabolic disorders increase the risk of DILI during ATT. This study aimed to identify risk factors for DILI, particularly metabolic disorders, during ATT.

**Methods:**

A multicenter prospective observational cohort study to evaluate adverse events during ATT was conducted in Korea from 2019 to 2021. Drug-susceptible patients with TB who had been treated with standard ATT for 6 months were included. The patients were divided into 2 groups depending on the presence of 1 or more metabolic conditions, such as insulin resistance, hypertension, obesity, and dyslipidemia. We monitored ATT-related adverse events, including DILI, and treatment outcomes. The incidence of DILI was compared between individuals with and without metabolic disorders, and related factors were evaluated.

**Results:**

Of 684 patients, 52 (7.6%) experienced DILI, and 92.9% of them had metabolic disorders. In the multivariable analyses, underlying metabolic disorders (adjusted hazard ratio [aHR], 2.85; 95% CI, 1.01–8.07) and serum albumin <3.5 g/dL (aHR, 2.26; 95% CI, 1.29–3.96) were risk factors for DILI during ATT. In the 1-month landmark analyses, metabolic disorders were linked to an elevated risk of DILI, especially significant alanine aminotransferase elevation. The treatment outcome was not affected by the presence of metabolic disorders.

**Conclusions:**

Patients with metabolic disorders have an increased risk of ATT-induced liver injury compared with controls. The presence of metabolic disorders and hypoalbuminemia adversely affects the liver in patients with ATT.

Unpredictable drug-induced liver injury (DILI) is one of the most common and challenging clinical problems for physicians. Diagnosis of DILI can be challenging as it mostly occurs in an idiosyncratic manner. Only a few patients develop DILI, which is not dose-related, occurs randomly within a few days to weeks, and has no novel biomarkers [1–3]. The consequences of DILI vary from mild liver enzyme elevation to hospitalization or even acute hepatic failure requiring liver transplantation. Antituberculosis (anti-TB) drugs are one of the most prominent causative drugs in the Asia-Pacific region, accounting for >20% of DILI cases [4].

Pulmonary TB, caused by *Mycobacterium tuberculosis* (MTB), poses an enormous socioeconomic burden. Over 1.6 million people died from TB in 2021, making it the 13^th^ leading cause of death worldwide [5]. Despite unremitting efforts to eradicate TB, it has not been properly controlled in many countries, including South Korea [6]. Although successful completion of anti-TB treatment (ATT) is the key to treatment, nonadherence is a huge hurdle, and DILI is one of the main factors contributing to nonadherence (10%–15% during the ATT) [7]. DILI also hinders treatment success rates because of discontinuation of medication, suboptimal medication dose due to elevated liver enzymes, or even changes in regimen to nonhepatotoxic secondary drugs.

However, the pathogenesis of DILI remains unclear. When genetically and environmentally susceptible patients are exposed to particular drugs, the modification of drug metabolism or inflammatory immune system activation can cause liver damage [8]. Drug-related risk factors include drugs undergoing liver metabolism, the production of reactive metabolites, and potential drug–drug interactions. Isoniazid (INH), ethambutol (EMB), rifampin (RIF), and pyrazinamide (PZA) inevitably carry a higher risk of DILI as they are metabolized, and their toxic intermediates accumulate in the liver [9]. Underlying metabolic risks, female sex, old age, alcohol intake, and underlying liver disease are also considered host-related risk factors for DILI. Furthermore, TB infection can modify the host's metabolic processes, raising concerns that patients with preexisting metabolic disorders may be more susceptible to DILI [10]. However, real-world data on whether the risk of DILI differs based on the presence of metabolic disorders are lacking.

Given the relevant practical needs, this study aimed to evaluate the association between metabolic disorders and DILI during ATT for the first time.

## METHOD

### Study Design and Population

**Figure 1.**
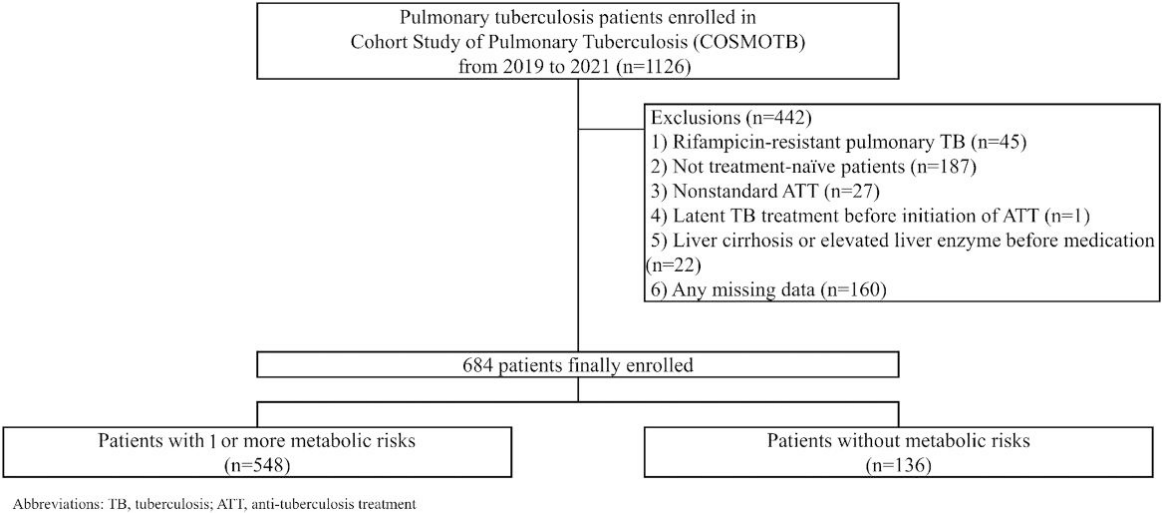
Flowchart of patient enrollment. Abbreviations: ATT, antituberculosis treatment; TB, tuberculosis.

The data were extracted from the Cohort Study of Pulmonary Tuberculosis (COSMOTB), a prospective observational cohort study conducted between 2019 and 2021 in 18 university-affiliated hospitals in South Korea [11]. This study prospectively and comprehensively collected data on treatment outcomes and adverse events, including DILI. We extracted data from the first 6 months of ATT. A total of 1126 patients were enrolled in the COSMOTB study. We excluded patients (1) who had RIF-resistant TB (n = 45); (2) who were not ATT naïve (n = 187); (3) who received nonstandard ATT (standard ATT was as follows: 2 months of intensive treatment [HREZ] + 4 months of continuation therapy [HRE]) (n = 27); (4) who were receiving latent TB treatment before ATT (n = 1); (5) who had baseline moderate to severe liver cirrhosis, aspartate aminotransferase (AST) >120 U/L, alanine aminotransferase (ALT) >120 U/L, or total bilirubin >1.5 mg/dL (n = 22); and (6) who had missing data regarding metabolic diseases or liver function (n = 160) (Figure 1). This study was approved by the Institutional Review Board of the Catholic University of Korea (IRB No. C19ONDI0458), and informed consent was obtained from all participants according to the study protocol.

### Follow-up and Data Collection

Demographic, socioeconomic, and clinical data were prospectively collected from enrolled patients using a case report form upon entry into the study. Baseline demographic variables included sex, age, body mass index, waist circumference, alcohol consumption (regular average consumption of 40 g/d for males and 20 g/d for females), smoking, and comorbidities. All patients underwent mycobacterial tests (chest imaging, acid-fast bacilli smear, mycobacterial culture, nucleic acid amplification test for the MTB complex, Xpert MTB/RIF assay, and drug susceptibility test). Baseline biochemical tests included white blood cells, hemoglobin, platelets, AST, ALT, total bilirubin, albumin, protein, creatinine, blood urea nitrogen, total cholesterol, triglyceride, high-density lipoprotein (HDL) cholesterol, low-density lipoprotein cholesterol, fasting glucose, hemoglobin A1C, hepatitis B surface antigen, and hepatitis C virus antibody. During follow-up, patients underwent several tests, including liver function tests (at 14 days, 28 days, 2 months, 3 months, 4 months, 5 months, and 6 months after ATT). Adverse drug reactions and medication compliance were assessed at every regular clinical visit using a predefined checklist. All patients were followed up until 1 of the following conditions was met: completion or discontinuation of ATT, death, or the last hospital visit.

### Metabolic Disorders

We defined patients with metabolic disorders as having at least 1 of the following conditions at enrollment [12]: (1) insulin resistance was defined as having a previous diagnosis of diabetes with or without diabetes medication, fasting serum glucose level of ≥126 mg/dL, or hemoglobin A1C >5.6%; (2) hypertension was defined as having a prior diagnosis of hypertension with or without medication, systolic blood pressure ≥130 mmHg, or diastolic blood pressure ≥85 mmHg; (3) dyslipidemia was defined as having a previous diagnosis of dyslipidemia, total cholesterol ≥240 mg/dL, triglycerides ≥150 mg/dL, or HDL cholesterol <40 mg/dL for males and 50 mg/dL for females; (4) obesity was defined as having a waist circumference ≥90 cm for males and ≥80 cm for females or a body mass index ≥25 kg/m^2^.

### Study Outcomes

The primary outcome was the identification of the relationship between metabolic disorders and DILI during ATT. DILI was defined as meeting 1 of the following, whichever came first [3, 13]: (1) ALT ≥200 U/L or ALT ≥120 U/L with hepatitis symptoms such as nausea, vomiting, epigastric discomfort, abdominal pain, anorexia; (2) AST ≥200 U/L or AST ≥120 U/L with hepatitis symptoms; (3) total bilirubin ≥3.0 mg/dL or total bilirubin ≥1.5 mg/dL with hepatitis symptoms. We also evaluated the favorable outcomes of ATT according to metabolic disorders, such as cure (bacteriologically defined smear- or culture-negative in the last month of ATT with at least once more previously) or treatment completion (completing treatment without evidence of treatment failure, which is defined as smear- or sputum-positive at the fifth month of ATT or later but without records of smear- or culture-negative in the last month of ATT with at least once more previously, either unavailable or incomplete test). Unfavorable outcomes were defined as a prolonged ATT of >6 months due to any cause, loss to follow-up, or death during ATT.

### Statistical Analysis

Data were summarized as the mean ± SD for continuous variables and number with percentage for categorical variables. The *t* test or Mann-Whitney *U* test was applied for continuous variables, and the chi-square or Fisher exact test was applied for categorical variables. Changes in liver enzymes during ATT were analyzed using a random coefficient model. The cumulative incidence of DILI was estimated using the Kaplan-Meier method and compared using the log-rank test. We used multivariable Cox proportional hazards models to identify the risk factors for DILI in patients with ATT. To evaluate early-onset liver enzyme elevation events within the first 35 days of ATT, we analyzed data from the second clinical visit after enrollment. We also analyzed patients who had at least 35 days of follow-up without liver enzyme elevation to determine the cumulative effect of ATT on liver injury. Furthermore, propensity score (PS) matching was conducted using age, sex, and viral hepatitis as variables using the nearest-neighbor method (1:1 matching with a caliper size of 0.1). A *P* value of <.05 was considered statistically significant. Statistical analyses were conducted using R software, version 4.1.3.

## RESULT

### Clinical Characteristics of the Study Population

The study population was comprised of 684 patients with pulmonary TB who initially received the standard ATT. The baseline characteristics of the patients are described in Table 1. The mean age was 59.9 ± 17.5 years, and 63.0% were male. A total of 548 (80.1%) participants had at least 1 metabolic disorder. Hypertension or high blood pressure (67.7%) was the most common metabolic disorder, followed by insulin resistance (66.6%), dyslipidemia (41.1%), and obesity (19.2%). The patients with metabolic disorders were older (62.9 ± 16.2 vs 47.8 ± 17.6; *P* < .001), consumed more alcohol (17.5% vs 9.6%; *P* = .032), and had a higher Charlson comorbidity index (CCI; 1.3 ± 1.4 vs 0.3 ± 0.9; *P* < .001). The prevalence of viral hepatitis did not differ between the 2 groups. The proportion with extrapulmonary organ involvement among all the study participants was 10.1%, and none of them had liver involvement. Favorable outcomes of ATT were not significantly different between the 2 groups (without metabolic disorders group [78.5%] vs with metabolic disorders group [71.8%]; *P* = .152).

**Table 1.** Clinical Characteristics of the Study Population

	Total	Without Metabolic Disorders^a^	With Metabolic Disorders^a^	*P* Value
	(n = 684)	(n = 136)	(n = 548)	
Demographic characteristics	*…*	…	…	…
Age, y	59.9 ± 17.5	47.8 ± 17.6	62.9 ± 16.2	<.001
Male sex, No. (%)	431 (63.0)	81 (59.6)	350 (63.9)	.405
Smoking, No. (%)	216 (31.6)	44 (32.4)	172 (31.4)	.909
Alcohol, No. (%)	109 (15.9)	13 (9.6)	96 (17.5)	.032
Body mass index, kg/m^2^	21.8 ± 3.6	20.4 ± 2.8	22.2 ± 3.7	<.001
Charlson comorbidity index	1.1 ± 1.4	0.3 ± 0.9	1.3 ± 1.4	<.001
Hepatitis B virus, No. (%)	22 (3.2)	3 (2.2)	19 (3.5)	.635
Hepatitis C virus, No. (%)	7 (1.0)	1 (0.7)	6 (1.1)	>.999
Metabolic disorders	*…*	…	…	…
No. of metabolic disorders	…	…	…	…
1	198 (28.9)	…	198 (36.1)	…
2	208 (30.4)	…	208 (38.0)	…
3	116 (17.0)	…	116 (21.2)	…
4	26 (3.8)	…	26 (4.7)	…
Diabetes	365 (53.4)	…	365 (66.6)	…
Hypertension	371 (54.2)	…	371 (67.7)	…
Central obesity	105 (15.4)	…	105 (19.2)	…
Imbalance of lipid profile	225 (32.9)	…	225 (41.1)	…
Laboratory findings	*…*	…	…	
White blood cell, 10³/μL	7.7 ± 3.9	7.2 ± 2.1	7.8 ± 4.2	.019
Hemoglobin, g/dL	12.7 ± 2.1	13.3 ± 1.8	12.6 ± 2.1	<.001
Platelets, 10³/μL	292.5 ± 115.1	303.1 ± 121.5	289.9 ± 113.4	.233
Protein, g/dL	7.1 ± 3.9	7.1 ± 0.7	7.2 ± 4.4	.773
Albumin, g/dL	3.9 ± 0.7	4.1 ± 0.6	3.9 ± 0.7	.010
AST, U/L	25.7 ± 12.7	24.2 ± 13.8	26.1 ± 12.3	.116
ALT, U/L	21.1 ± 15.0	20.2 ± 15.4	21.3 ± 14.9	.405
Total bilirubin, mg/dL	0.6 ± 0.3	0.6 ± 0.2	0.6 ± 0.3	.804
Creatinine, mg/dL	1.0 ± 4.2	1.5 ± 9.4	0.9 ± 0.6	.425
Blood urea nitrogen, mg/dL	15.1 ± 8.0	12.6 ± 5.0	15.8 ± 8.5	<.001
Glucose, mg/dL	130.6 ± 62.2	100.0 ± 11.6	137.0 ± 66.5	<.001
Hemoglobin A1C, %	6.7 ± 1.9	5.4 ± 0.1	6.8 ± 1.9	<.001
Total cholesterol, mg/dL	159.1 ± 42.5	171.3 ± 35.5	156.9 ± 43.3	.003
Low-density lipoprotein, mg/dL	91.5 ± 32.7	94.9 ± 20.3	91.2 ± 33.5	.413
High-density lipoprotein, mg/dL	47.4 ± 16.5	59.7 ± 12.0	46.4 ± 16.5	<.001
Triglyceride, mg/dL	119.0 ± 76.9	81.8 ± 28.5	123.1 ± 79.5	<.001
Tuberculosis characteristics	*…*	…	…	
Extrapulmonary involvement, No. (%)	69 (10.1)	13 (9.6)	56 (10.2)	.944
Drug-induced liver injury	…	…	…	
Overall, No. (%)	52 (7.6)	4 (2.9)	48 (8.8)	.035
ALT, No. (%)	28 (4.1)	2 (1.5)	26 (4.7)	.138
AST, No. (%)	23 (3.4)	2 (1.5)	21 (3.8)	.271
Total bilirubin, No. (%)	26 (3.8)	3 (2.2)	23 (4.2)	.403
Treatment outcome	…	…	…	
Favorable (cure or treatment complete), No. (%)	478 (73.1)	102 (78.5)	376 (71.8)	.152

Abbreviations: ALT, alanine aminotransferase; AST, aspartate aminotransferase; ATT, antituberculosis treatment.

a Metabolic disorders were defined as having 1 or more of the following conditions: insulin resistance, hypertension, obesity, and dyslipidemia.

### Drug-Induced Liver Injury During Antituberculosis Treatment

**Figure 2.**
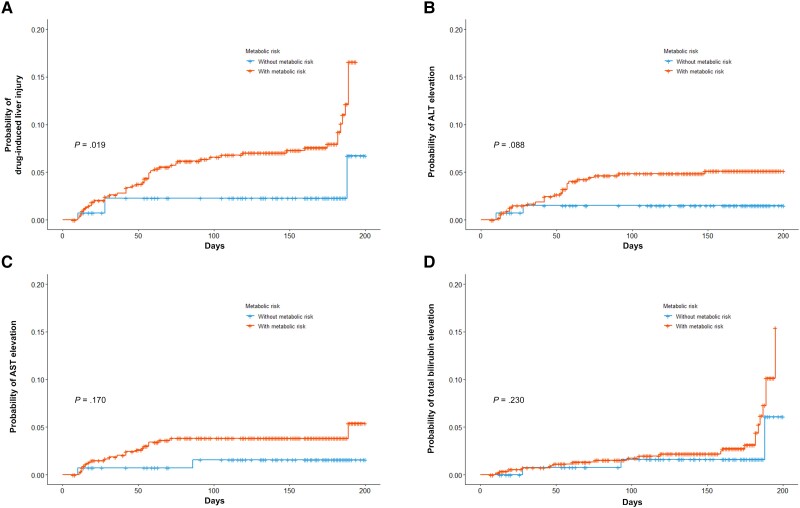
Cumulative risks of drug-induced liver injury during antituberculosis treatment according to the presence of metabolic disorders. *A*, Drug-induced liver injury. *B*, Clinically significant alanine aminotransferase elevation. *C*, Clinically significant aspartate aminotransferase elevation. *D*, Clinically significant total bilirubin elevation. Abbreviations: ALT, alanine aminotransferase; AST, aspartate aminotransferase.

**Figure 3.**
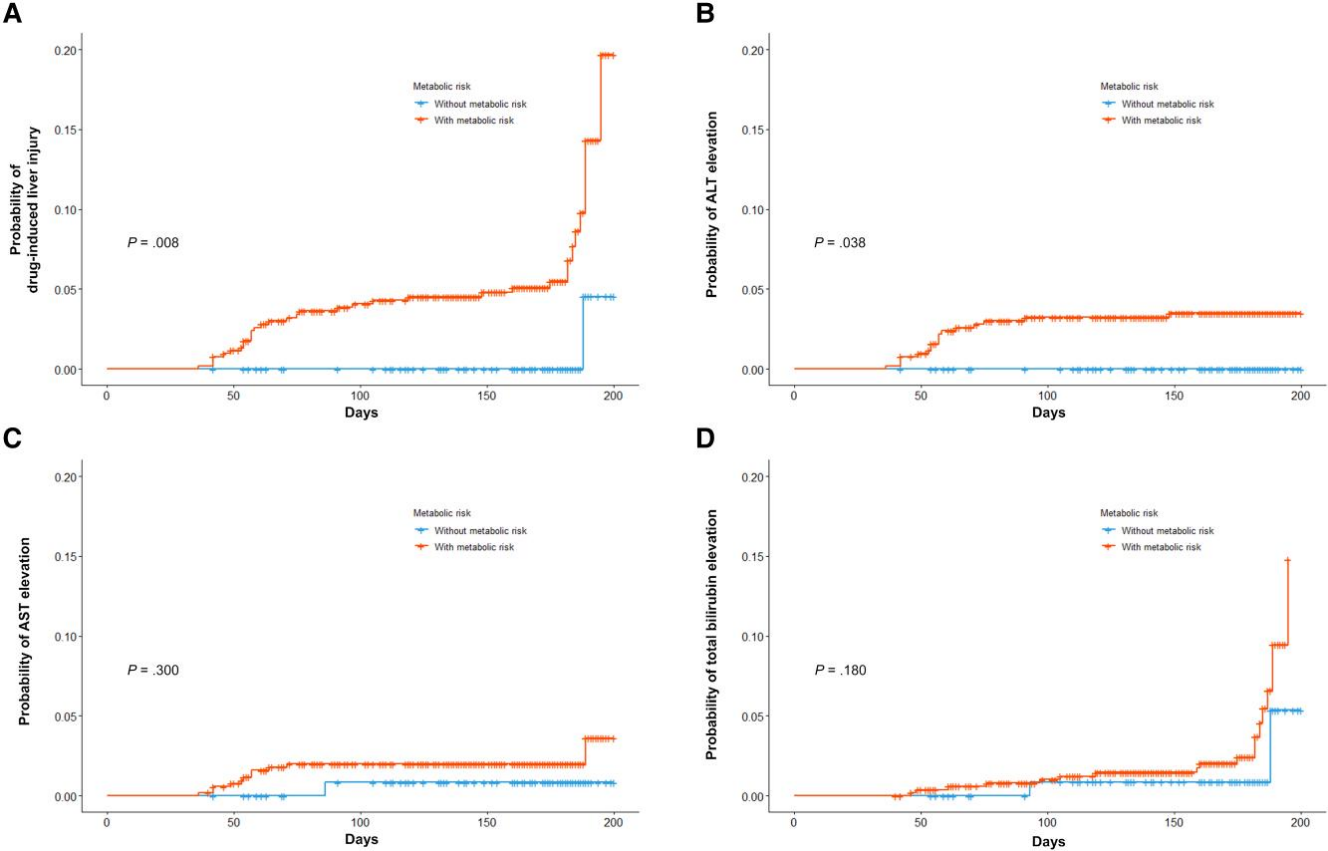
One-month landmark analysis for cumulative risks of drug-induced liver injury during antituberculosis treatment according to the presence of metabolic disorders. *A*, Drug-induced liver injury. *B*, Clinically significant alanine aminotransferase elevation. *C*, Clinically significant aspartate aminotransferase elevation. *D*, Clinically significant total bilirubin elevation. Abbreviations: ALT, alanine aminotransferase; AST, aspartate aminotransferase.

Scatter plots of the repeated measurements of liver enzymes over 6 months are shown in Supplementary Figure 1. Outliers were observed more frequently in the metabolic disorder group. However, the results of the random coefficient model were not significantly different between the 2 groups. Fifty-two patients (7.6%) developed DILI during the study period. The median onset of DILI (range) was 7.9 (4.0–18.0) weeks. There were 28 (4.1%), 23 (3.4%), and 26 (3.8%) patients with clinically significant elevations in ALT, AST, and total bilirubin levels during ATT. The metabolic disorders group showed more DILI than the nonmetabolic disorders group (8.8% vs 2.9%; *P* = .035) (Table 1). Cumulative incidence rates are presented in Supplementary Table 1. A significantly higher cumulative incidence of DILI was observed in patients with metabolic disorders than in those without (log-rank *P* = .019) (Figure 2*A*). For patients with metabolic disorders, the percentage with clinically significant ALT elevation was higher than that of their non–clinically significant counterparts (*P* = .088) (Figure 2*B*). The rates of AST and bilirubin elevation were insignificant but were numerically higher in the metabolic disorder group (Figure 2*C* and *D*). Liver enzyme changes in the ALT, AST, and total bilirubin levels within 1 month did not differ between the 2 groups (Supplementary Figure 2). A total of 635 patients in this study had at least 1 month of observation without liver enzyme elevation. Therefore, we further conducted the 1-month landmark analyses. In the 1-month landmark analyses, the cumulative incidence rates of total DILI (log-rank *P* = .008) (Figure 3*A*) and ALT (log-rank *P* = .034) (Figure 3*B*) were significantly higher in the metabolic disorder group than in their counterpart. AST and total bilirubin in the 1-month landmark analyses were higher but were not statistically significant (Figure 3*C* and *D*). In the PS-matched cohort, the group with metabolic disorders had a higher risk of ALT elevation (log-rank *P* = .044) (Supplementary Figure 3) but not of other factors, such as AST or total bilirubin.

### Evaluation of the Risk Factors for Drug-Induced Liver Injury According to Metabolic Disorders

Supplementary Table 2 shows the characteristics of the DILI group (n = 52) compared with the non-DILI group (n = 632). Compared with their counterparts, the patients with DILI had higher metabolic risk (92.3% vs 79.1%; *P* = .035). They had lower levels of serum albumin than those without DILI at baseline (3.7 ± 0.7 vs 4.0 ± 0.7; *P* = .031). Univariate and multivariate Cox regression analyses were performed to evaluate the risk of DILI. In the univariate analyses, the following variables were selected because of their associations with the risk of DILI: metabolic disorders, age (≥60 years), male sex, smoking, alcohol consumption, CCI score, white blood cell count, hemoglobin level, platelet count, protein and albumin levels, creatinine, baseline ALT, AST, and total bilirubin levels, chronic hepatitis B or C, and extrapulmonary involvement. Metabolic disorders (hazard ratio [HR], 3.17; 95% CI, 1.14–8.79; *P* = .027) and serum albumin level (<3.5 g/dL; HR, 2.36; 95% CI, 1.35–4.12; *P* = .003) were associated with DILI in univariable analyses. Metabolic disorders, old age (≥60 years), sex, and serum albumin level (<3.5 g/dL) were selected as variables using the forward conditional method in multivariable analyses. In adjusted analyses, having a metabolic disorder was identified as a risk factor for DILI (adjusted HR [aHR], 2.85; 95% CI, 1.01–8.07; *P* = .042), along with serum albumin <3.5 g/dL (aHR, 2.26; 95% CI, 1.29–3.96; *P* = .005) (Table 2).

**Table 2.** Risk Factors Associated With Drug-Induced Liver Injury During Antituberculosis Treatment

	Univariate Analysis		Multivariate Analysis	
	HR (95% CI)	*P* Value	HR (95% CI)	*P* Value
Metabolic disorders^a^	3.17 (1.14–8.79)	.027	2.85 (1.01–8.07)	.042
Age, ≥60 y	1.41 (0.81–2.47)	.225	1.06 (0.59–1.89)	.850
Male sex	0.83 (0.48–1.44)	.506	0.77 (0.44–1.34)	.357
Smoking	0.96 (0.53–1.73)	.885	…	…
Alcohol	1.63 (0.85–3.10)	.140	…	…
Charlson comorbidity index	0.91 (0.73–1.14)	.427	…	…
White blood cells, >10 × 10³/μL	1.10 (0.54–2.26)	.793	…	…
Hemoglobin, <12 g/dL	1.18 (0.67–2.09)	.569	…	…
Platelets, <150 × 10³/μL	0.91 (0.28–2.91)	.871	…	…
Protein, <6 g/dL	0.55 (0.24–1.22)	.143	…	…
Albumin, <3.5 g/dL	2.36 (1.35–4.12)	.003	2.26 (1.29–3.96)	.005
AST, >40 U/L	1.60 (0.75–3.40)	.220	…	…
ALT, >40 U/L	0.55 (0.17–1.78)	.322	…	…
Total bilirubin, >1.2 mg/dL	0.58 (0.08–4.24)	.596	…	…
Creatinine, >1.4 mg/dL	0.59 (0.08–4.25)	.597	…	…
HBV, HCV infection	1.39 (0.43–4.48)	.576	…	…
Extrapulmonary involvement	0.69 (0.361–1.54)	.366	…	…

Abbreviations: ALT, alanine aminotransferase; AST, aspartate aminotransferase; HBV, hepatitis B virus; HCV, hepatitis C virus; HR, hazard ratio.

a Metabolic disorders were defined as having 1 or more of the following conditions: insulin resistance, hypertension, obesity, and dyslipidemia.

## DISCUSSION

In this multicenter cohort study of TB, we found that 7.6% of patients (52 out of 684 patients) had a DILI during ATT. Among them, 92.9% had 1 or more metabolic disorders, such as insulin resistance, hypertension, obesity, or dyslipidemia. Patients with metabolic disorders have a 2.85-fold higher risk of developing DILI. None of the subjects without metabolic disorders experienced ALT elevation 1 month after ATT. In addition, serum albumin <3.5 g/dL at baseline was also found to exacerbate liver injury. In previous studies, the incidence of DILI was reported as 3.0%–7.3% [2, 14–16]. Most liver injury cases have been reported to occur in the early treatment period, with a mean time of 3.4 weeks in China, a median of 1 month in the United Kingdom, and a mean time of 1.9 months in India [16–18]. Hepatitis symptoms at the onset of liver enzyme elevation were seen in 65.4% of the patients in the current study, similar to a previous study [14].

The effect of metabolic disorders on DILI is unclear but highly suspicious [4, 19]. In our study, metabolic disorders significantly increased the risk of DILI during ATT. There are several possible explanations for this. First, patients with metabolic disorders are exposed to oxidative stress, which increases cellular oxidants and lipid peroxidation and depletes antioxidants [19, 20]. When drugs such as INH, which produces intracellular oxidants during metabolism, are administered, patients with metabolic disorders are more susceptible to liver injury than those without metabolic disorders [19, 21]. Second, metabolic disorders can cause mitochondrial dysfunction [20]. Stressed mitochondria can trigger hepatic necrosis when damaged by extrinsic factors, such as ATT [19, 22]. INH, PZA, and RIF are all known to have mitochondrial toxicity [23]. Third, metabolic disorders alter the expression of hepatic transporters, which play a critical role in hepatocyte metabolic processes [19, 24]. They cause cholestasis and free bile acid salt congestion in hepatocytes. RIF and rifabutin can exacerbate the disruption of hepatic transporters and cause liver injury [25]. Finally, pro-inflammatory conditions caused by metabolic disorders contribute to the overactivation of the immune-inflammatory system [19, 26]. The immune response often determines the severity of liver injury, and an imbalance in the immunoinflammatory system exacerbates hepatocyte damage [8].

We found that a serum albumin level of <3.5 g/dL also increased the risk of DILI. Albumin reflects general health conditions, including nutritional status, and is affected by chronic liver disease, kidney disease, severe infections, and inflammation. These medical conditions decrease drug clearance, resulting in drug levels exceeding the threshold dose [19]. They also induce a chronic inflammatory state that facilitates cellular senescence [19]. A previous study in India also showed that low serum albumin levels were a risk factor for DILI [27]. Moreover, the serum albumin level at the onset of DILI is associated with disease severity and prognosis [3]. Indeed, when the albumin level before ATT is <3.5 mg/dL, complete liver biochemical tests and ultrasonography are recommended [2]. In previous studies, old age, female sex, alcohol intake, and low body weight were reported to be associated with DILI; however, no common risk factors were found [14, 16, 18]. In contrast, none of these factors increased the risk of liver injury in the present study. These findings should be confirmed in future studies.

Most DILI cases present a hepatocellular pattern with a dominant elevation in aminotransferase levels. Naturally, bilirubin elevation follows ALT or AST elevation with a lag time of up to 4 weeks [28]. Moreover, hepatocellular injury can be accompanied by secondary intrahepatic cholestasis. These findings explain the late onset of bilirubin elevation observed in the present study.

To monitor adverse effects, it is recommended to measure serum transaminases and bilirubin levels in all adults beginning treatment for TB disease. Generally, fortnightly or monthly testing and examinations by a physician are sufficient for adult patients with drug-susceptible pulmonary TB who have minimal baseline abnormalities and tolerate treatment. The US guidelines recommend performing liver function tests only at baseline unless there are abnormalities at baseline, symptoms consistent with hepatotoxicity develop, or the patient has other risk factors associated with hepatotoxicity or prior DILI [29]. Based on our study results, we recommend considering patients with metabolic disorders to be potentially at risk of DILI. We recommend that these patients be closely monitored for hepatotoxicity-related symptoms and signs and undergo regular liver function tests until they have completed 2 months of ATT. Further research is necessary to assess the impact of regular liver function tests on the development of DILI to expand strategies to monitor DILI. In addition, it is essential to develop and modify strategies to monitor DILI based on the local health care infrastructure and systems.

This study has several limitations. First, nonalcoholic fatty liver disease was not assessed because abdominal imaging or liver biopsy was not included in this study protocol. However, the chance of having fatty liver in patients with metabolic disorders is >90% [30]. Second, repeated measurements of liver enzymes were not required because of the nature of the study. ALT, AST, and total bilirubin levels were measured repeatedly during patient visits or at the doctor's discretion. However, most patients regularly visited clinics and were monitored using a mixed private–public TB patient-monitoring program. Therefore, the unobserved events of abnormal liver enzyme elevation were theoretically or procedurally impossible, and all DILI cases were included in this analysis. In addition, we were not able to include conjugated bilirubin concentration or alkaline phosphatase, which reflected the liver injury pattern. The definition of DILI in our study differs from that of previous studies, but it is widely acceptable for ATT. In addition, the case definitions differ from those of a previous study [13].

In conclusion, DILI was found to be significantly increased in patients with metabolic disorders. In addition, low serum albumin levels were found to be associated with DILI. Considering the adverse events associated with DILI, recognition of these risk factors, close monitoring, and early intervention may be crucial for patients on ATT.

## Supplementary Material

ofad422_Supplementary_Data

## Supplementary Data

Supplementary materials are available at *Open Forum Infectious Diseases* online. Consisting of data provided by the authors to benefit the reader, the posted materials are not copyedited and are the sole responsibility of the authors, so questions or comments should be addressed to the corresponding author.
