# Supplementary material for: Metabolic Disorders Are Associated With Drug-Induced Liver Injury During Antituberculosis Treatment: A Multicenter Prospective Observational Cohort Study in Korea
Source: Open Forum Infect Dis. 2023 Aug 7;10(8):ofad422. doi: 10.1093/ofid/ofad422 (PMC10468151; doi:10.1093/ofid/ofad422)
Supplement: ofad422_Supplementary_Data [file ofad422_supplementary_data.docx]

**Supplementary materials**

**Table of contents**

- **Supplementary Fig. 1.** Scatter plots of aspartate aminotransferase, alanine aminotransferase, and total bilirubin by metabolic disorders
- **Supplementary Fig. 2.** Cumulative risks of liver enzyme elevation in patients with ATT according to the presence of metabolic disorders during the first month
- **Supplementary Fig. 3.** Cumulative risks of alanine aminotransferase (ALT) elevation in patients with anti-tuberculosis treatment based on the presence of metabolic disorders in propensity score matched cohort
- **Supplementary Table 1.** Cumulative incidence rate of drug-induced liver injury, alanine aminotransferase (ALT) elevation, aspartate aminotransferase (AST) elevation, and total bilirubin elevation during anti-tuberculosis treatment
- **Supplementary Table 2.** Baseline characteristics by the presence of drug-induced liver injury during anti-tuberculosis treatment

**Supplementary Fig. 1.** Scatter plots of (A) alanine aminotransferase (ALT), (B) aspartate aminotransferase (AST), and (C) total bilirubin by metabolic disorders


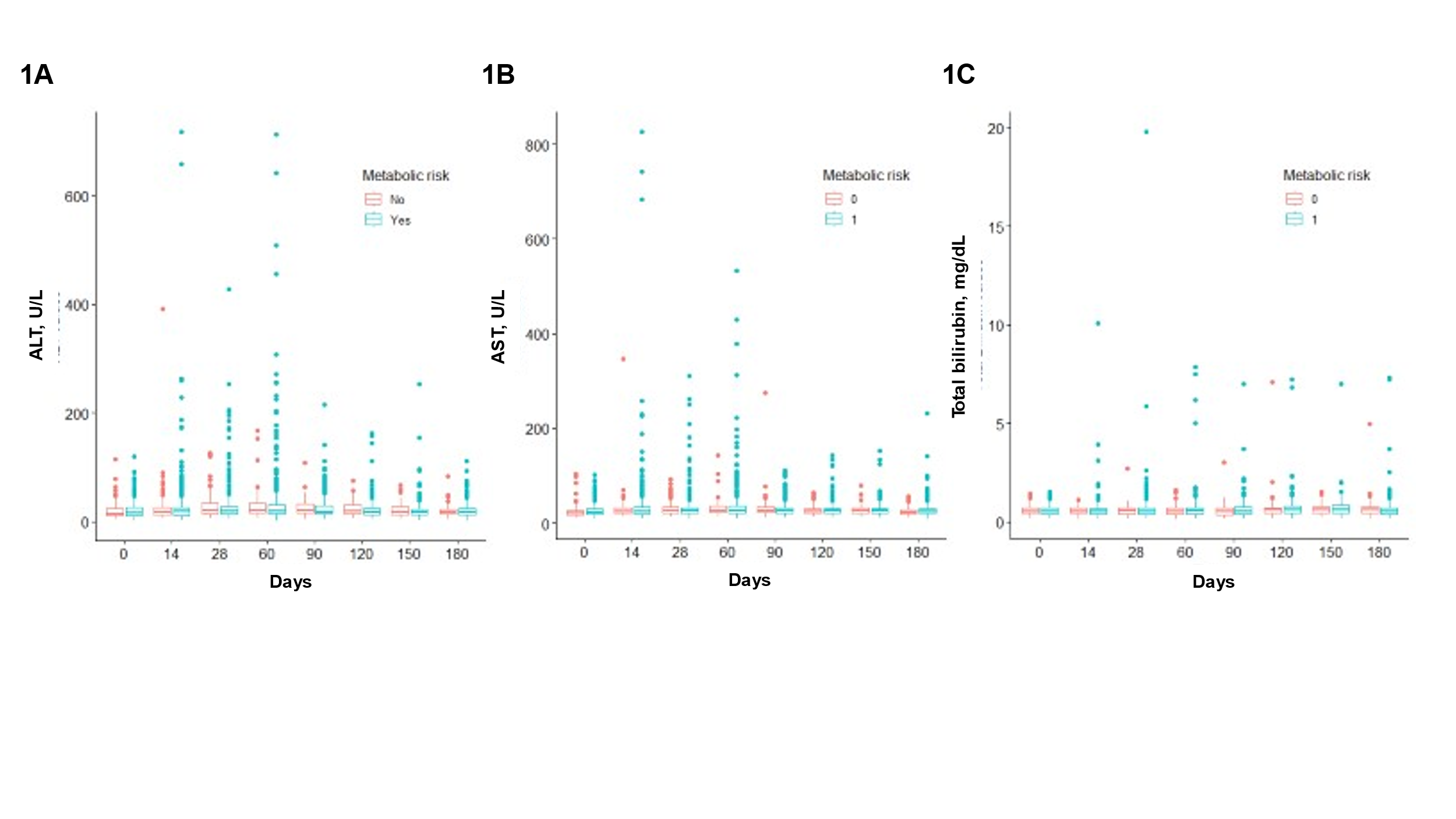


**Supplementary Fig. 2.** Cumulative risks of liver enzyme elevation in patients with ATT according to the presence of metabolic disorders during the first month

1. aspartate aminotransferase (ALT) elevation
2. alanine aminotransferase (AST) elevation
3. total bilirubin elevation


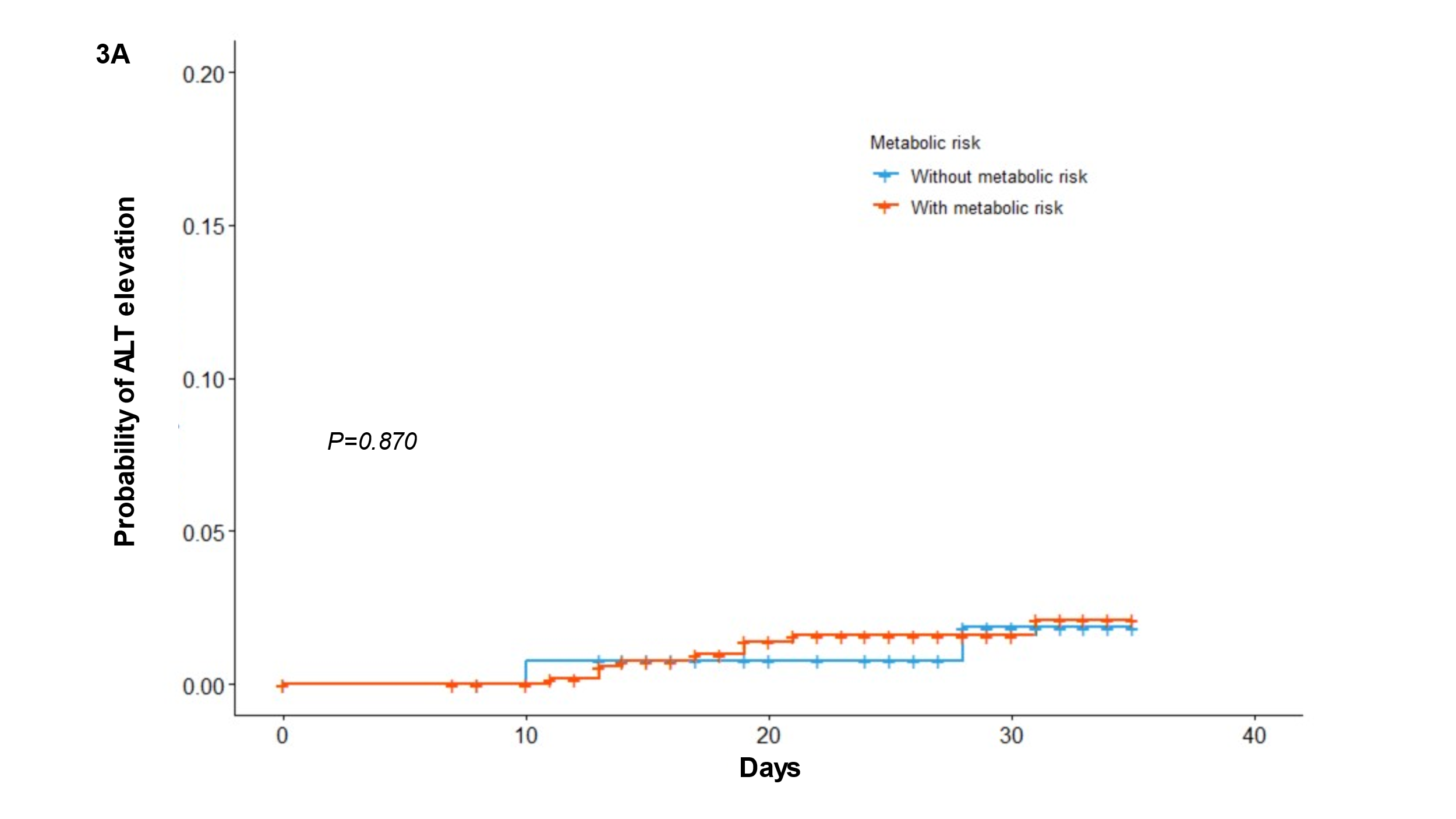


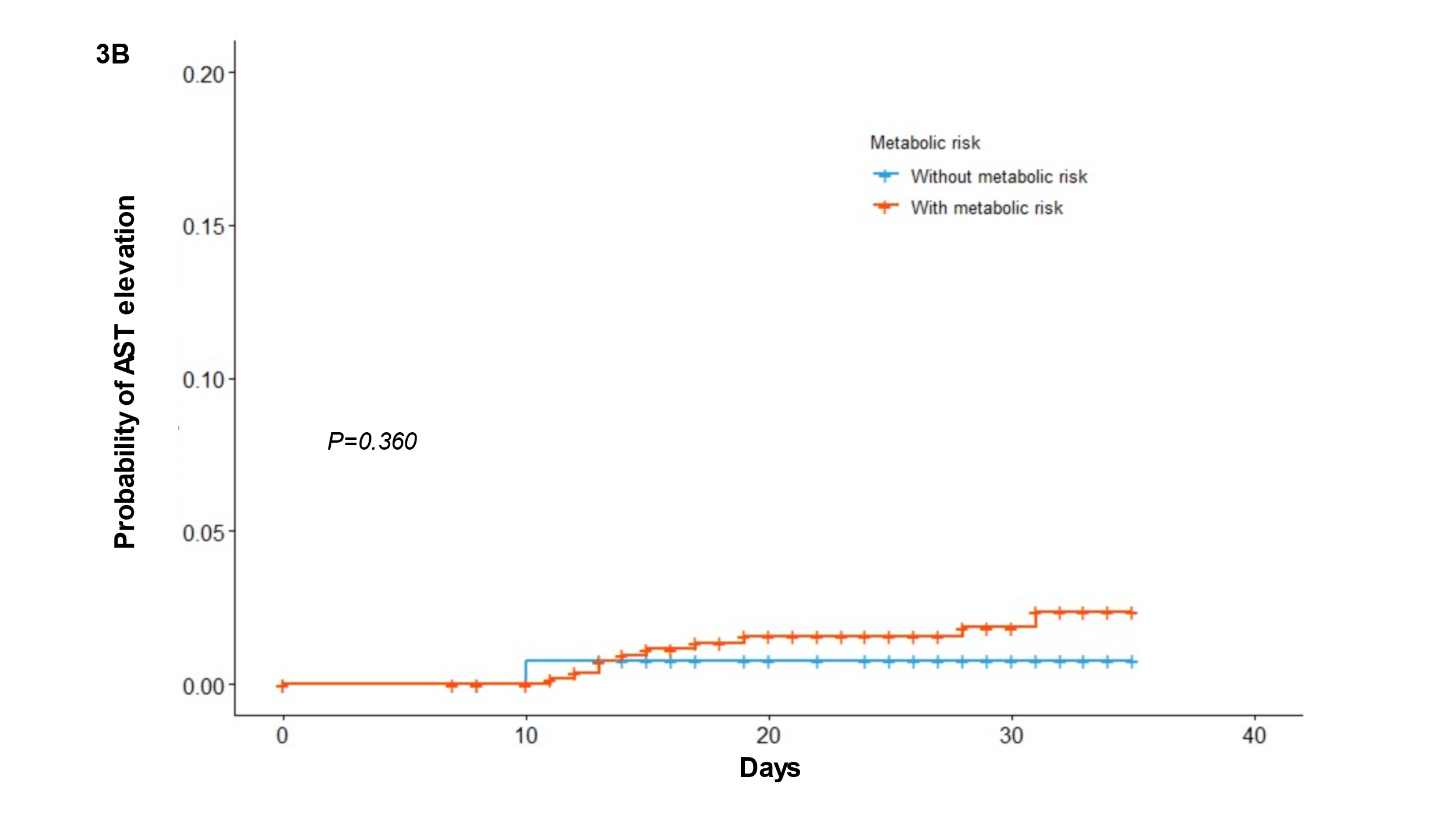


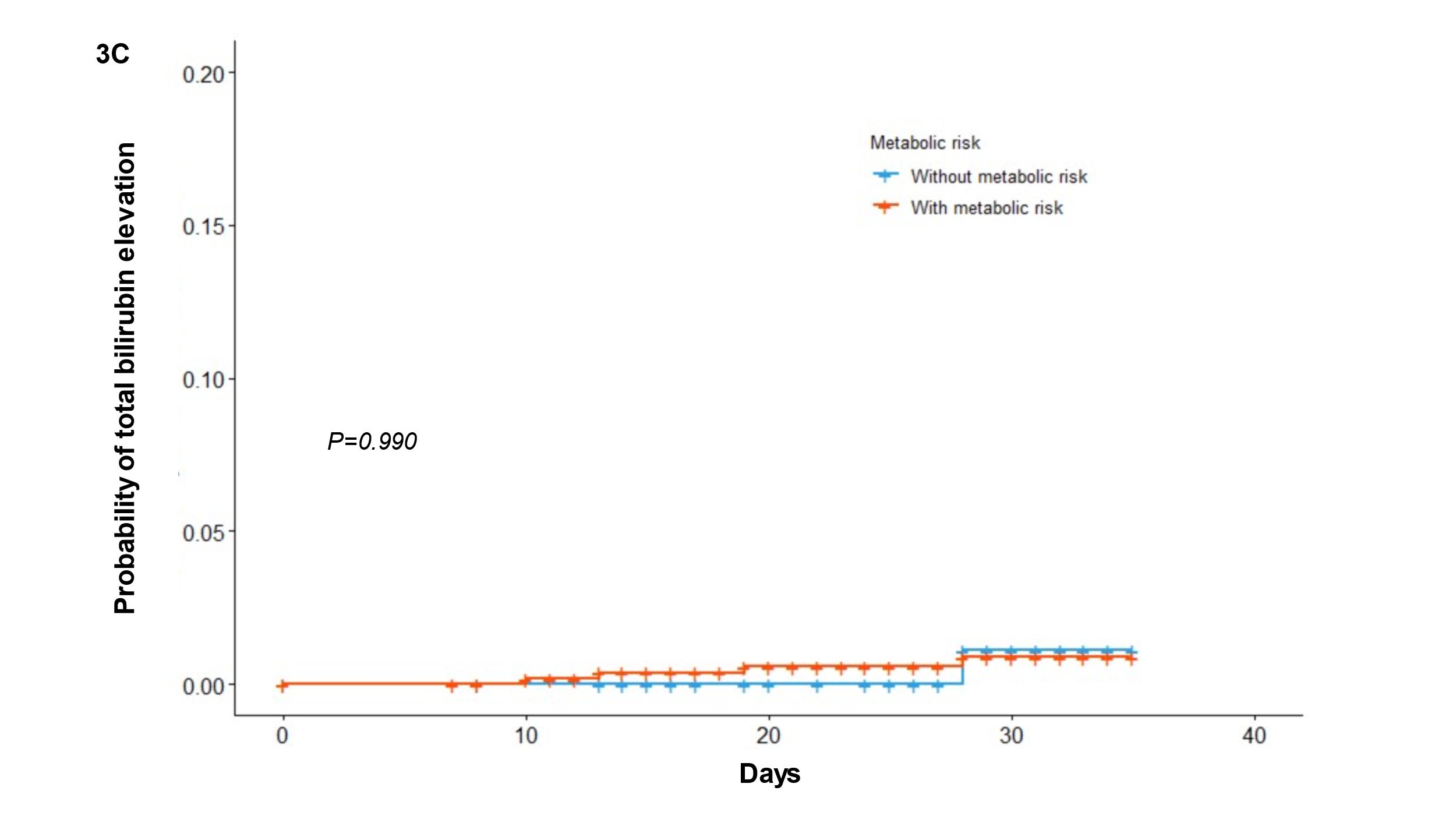


**Supplementary Fig. 3.** Cumulative risks of aspartate aminotransferase (ALT) elevation in patients with anti-tuberculosis treatment based on the presence of metabolic disorders in propensity score matched cohort


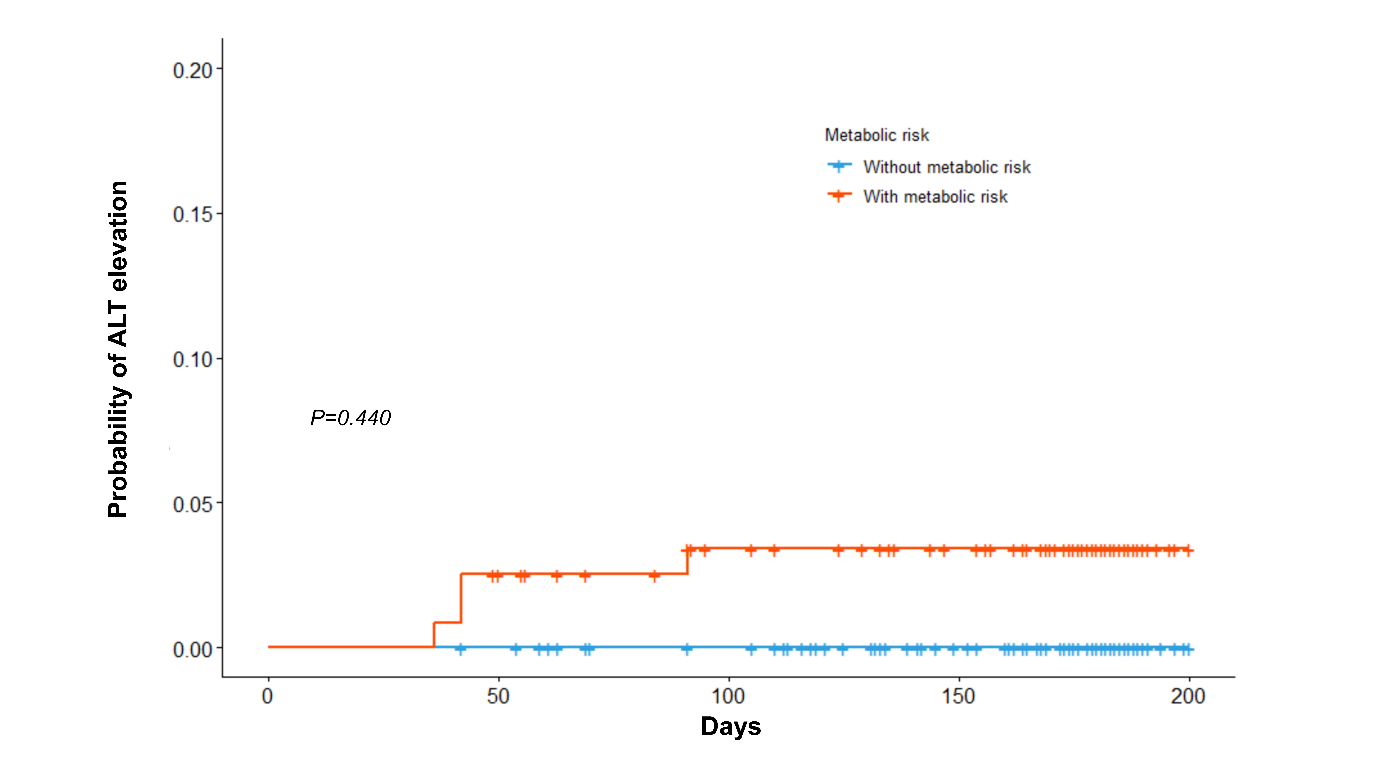


**Supplementary Table 1.** Cumulative incidence rate of drug-induced liver injury, alanine aminotransferase (ALT) elevation, aspartate aminotransferase (AST) elevation, and total bilirubin elevation during anti-tuberculosis treatment

| Treatment duration (month) | 1 | 2 | 3 | 4 | 5 | 6 |
| --- | --- | --- | --- | --- | --- | --- |
| Cumulative overall drug-induced liver injury incidence | | | | | | |
| *Entire study period* | | | | | | |
| Without metabolic disorders (%) | 2.3 | 2.3 | 2.3 | 2.3 | 2.3 | 2.3 |
| With metabolic disorders (%) | 2.4 | 5.3 | 6.4 | 7.0 | 7.3 | 9.2 |
| *One-month landmark analysis* | | | | | | |
| Without metabolic disorders (%) | - | 0.0 | 0.0 | 0.0 | 0.0 | 0.0 |
| With metabolic disorders(%) | - | 2.8 | 3.9 | 4.5 | 4.8 | 6.8 |
| Cumulative ALT elevation incidence | | | | | | |
| *Entire study period* | | | | | | |
| Without metabolic disorders (%) | 1.5 | 1.5 | 1.5 | 1.5 | 1.5 | 1.5 |
| With metabolic disorders (%) | 1.5 | 4.0 | 4.9 | 4.9 | 5.1 | 5.1 |
| *One-month landmark analysis* | | | | | | |
| Without metabolic disorders (%) | - | 0.0 | 0.0 | 0.0 | 0.0 | 0.0 |
| With metabolic disorders (%) | - | 2.4 | 3.2 | 3.2 | 3.5 | 3.5 |
| Cumulative AST elevation incidence | | | | | | |
| *Entire study period* | | | | | | |
| Without metabolic disorders (%) | 0.7 | 0.7 | 1.6 | 1.6 | 1.6 | 1.6 |
| With metabolic disorders (%) | 1.7 | 3.4 | 3.8 | 3.8 | 3.8 | 3.8 |
| *One-month landmark analysis* | | | | | | |
| Without metabolic disorders (%) | - | 0.0 | 0.8 | 0.8 | 0.8 | 0.8 |
| With metabolic disorders (%) | - | 1.6 | 2.0 | 2.0 | 2.0 | 2.0 |
| Cumulative total bilirubin elevation incidence | | | | | | |
| *Entire study period* | | | | | | |
| Without metabolic disorders (%) | 0.8 | 0.8 | 0.8 | 1.6 | 1.6 | 1.6 |
| With metabolic disorders (%) | 0.7 | 1.3 | 1.5 | 2.2 | 2.2 | 4.4 |
| *One-month landmark analysis* | | | | | | |
| Without metabolic disorders (%) | - | 0.0 | 0.0 | 0.9 | 0.9 | 0.9 |
| With metabolic disorders (%) | - | 0.6 | 0.8 | 1.5 | 1.5 | 3.7 |

Abbreviations: ALT, aspartate aminotransferase; AST, alanine aminotransferase; ATT, anti-tuberculosis treatment

**Table 2.** Baseline characteristics by the presence of drug-induced liver injury during anti-tuberculosis treatment

|  | Without DILI | With DILI | P-value |
| --- | --- | --- | --- |
|  | (N=632) | (N=52) |  |
| Age ≥ 60 years, n (%) | 59.6 ± 17.5 | 63.8 ± 18.3 | 0.092 |
| Male sex, n (%) | 401 (63.4%) | 30 (57.7%) | 0.498 |
| Smoking, n (%) | 200 (31.6%) | 16 (30.8%) | >0.999 |
| Alcohol, n (%) | 97 (15.3%) | 12 (23.1%) | 0.205 |
| Charlson comorbidity index | 1.1 ± 1.4 | 0.9 ± 1.1 | 0.321 |
| HBV or HCV infection, n (%) | 26 (4.1%) | 3 (5.8%) | 0.833 |
| Metabolic disorders^*^ | 500 (79.1%) | 48 (92.3%) | 0.035 |
| *Laboratory findings* |  |  |  |
| White blood cell, 10³/μL | 7.7 ± 4.0 | 7.3 ± 2.6 | 0.365 |
| Hemoglobin, g/dL | 12.7 ± 2.1 | 12.8 ± 2.3 | 0.871 |
| Platelets, 10³/μL | 292.6 ± 113.2 | 290.5 ± 137. | 0.916 |
| Protein, g/dL | 7.2 ± 4.1 | 6.9 ± 0.9 | 0.175 |
| Albumin, g/dL | 4.0 ± 0.7 | 3.7 ± 0.7 | 0.031 |
| AST, U/L | 25.7 ± 12.7 | 26.3 ± 12.2 | 0.739 |
| ALT, U/L | 21.2 ± 15.1 | 19.5 ± 12.5 | 0.408 |
| Total bilirubin, mg/dL | 0.6 ± 0.3 | 0.6 ± 0.3 | 0.331 |
| Creatinine, mg/dL | 1.0 ± 4.4 | 0.8 ± 0.4 | 0.311 |
| Extrapulmonary involvement, n (%) | 62 (9.8%) | 7 (13.5%) | 0.548 |

Abbreviations: ALT, aspartate aminotransferase; AST, alanine aminotransferase; DILI, drug-induced liver injury HBV, hepatitis B virus; HCV, hepatitis C virus

^*^Metabolic disorders was defined as having one or more following conditions: insulin resistance, hypertension, obesity, and dyslipidemia.
